# Supplementary material for: Identification of photoperiod-induced specific miRNAs in the adrenal glands of Sunite sheep (Ovis aries)
Source: Front Vet Sci. 2022 Jul 22;9:888207. doi: 10.3389/fvets.2022.888207 (PMC9354845; doi:10.3389/fvets.2022.888207)
Supplement: Supplementary file 1 [file Data_Sheet_1.ZIP › Supplementary file/miRDeep2 file.zip!/miRDeep2 file/Novel_Structure/SPLP42A1/Novel_380.pdf]

Provisional ID	:	NC_019471.2_20420
Score total	:	0.9
Score for star read(s)	:	-1.3
Score for read counts	:	0
Score for mfe	:	0.6
Score for randfold	:	1.6
Score for cons. seed	:	
Total read count	:	19
Mature read count	:	18
Loop read count	:	0
Star read count	:	1

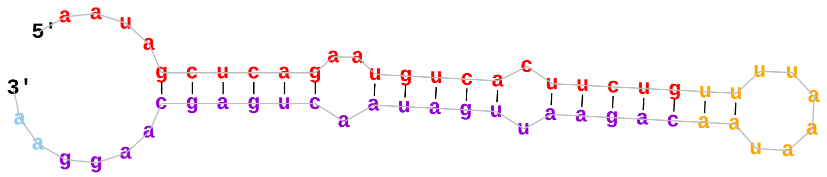

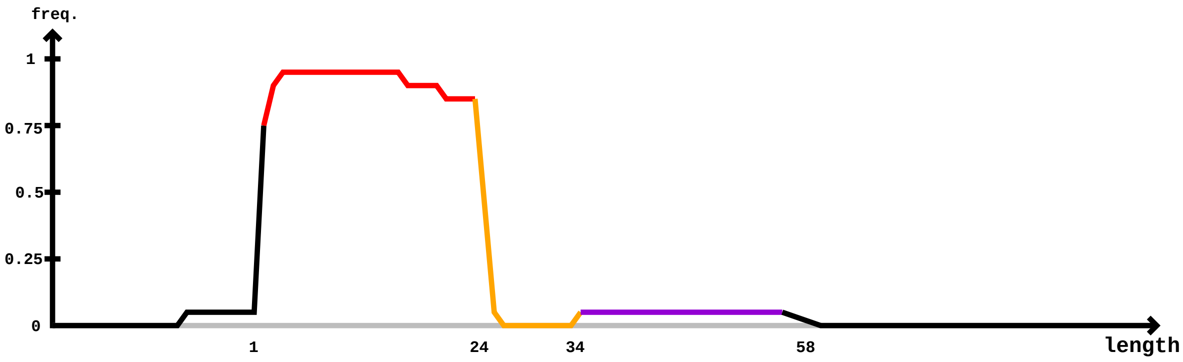

Mature Star

```
5'- uuuguaauucuuuacugugg aauagcucagaaugucacuucug uuuuaaauaa cagaauugauaacugagcaagg aaacguaauuuggauuauaaaauucuugcucuaauaaa   -3'   obs
```

```
5'- uuuguaauucuuuacuguggaauagcucagaaugucacuucuguuuuaaauaacagaauugauaacugagcaaggaaacguaauuuggauuauaaaauucuugcucuaauaaa -3'  obs
    uuuguaauucuuuacuguggaauagcucagaaugucacuucuguuuuaaauaacagaauugauaacugagcaaggaaacguaauuuggauuauaaaauucuugcucuaauaaa      exp
    ((((((((((.((((.((......((((((..(((((.(((((((......))))))).))))).))))))......))))))...))))))))))................. reads  mm  sample
    ............uacuguggaauagcucagaaugu.............................................................................. 1      0   seq
    ....................aauagcucagaaugucacu.......................................................................... 1      0   seq
    ....................aauagcucagaaugucacuucug...................................................................... 13     0   seq
    .....................auagcucagaaugucacuucug...................................................................... 3      0   seq
    ......................uagcucagaaugucacuucuguu.................................................................... 1      0   seq
    .....................................................cagaauugauaacugagcaagg...................................... 1      0   seq
```
